# The spin-dependent transport properties of zigzag α-graphyne nanoribbons and new device design

**DOI:** 10.1038/srep25914

**Published:** 2016-05-16

**Authors:** Yun Ni, Xia Wang, Wei Tao, Si-Cong Zhu, Kai-Lun Yao

**Affiliations:** 1 Wenhua College, Huazhong University of Science and Technology, Wuhan, China, 430074; 2 School of Science, Wuhan University of Science and Technology, Wuhan, China, 430081; 3 School of Physics and Wuhan National High Magnetic Field Center, Huazhong University of Science and Technology, Wuhan, China, 430074

## Abstract

By performing first-principle quantum transport calculations, we studied the electronic and transport properties of zigzag α-graphyne nanoribbons in different magnetic configurations. We designed the device based on zigzag α-graphyne nanoribbon and studied the spin-dependent transport properties, whose current-voltage curves show obvious spin-polarization and conductance plateaus. The interesting transport behaviours can be explained by the transport spectra under different magnetic configurations, which basically depends on the symmetry matching of the electrodes’ bandstructures. Simultaneously, spin Seebeck effect is also found in the device. Thus, according to the transport behaviours, zigzag α-graphyne nanoribbons can be used as a dual spin filter diode, a molecule signal converter and a spin caloritronics device, which indicates that α-graphyne is a promising candidate for the future application in spintronics.

Spintronics, a new type of electronics which concerns the interplay of spin and charge transport, has attracted great attention these years^1^,^2^. It is predicted that spintronics will be exploited for electronic applications and take the place of traditional silicon-based electronics^3^,^4^. Meanwhile, graphene, a single-layered two-dimensional crystal with honeycomb lattice structure, has been widely studied since the successful fabrication in 2004^5^. The electronic properties of graphene, such as high carrier mobility, long spin relaxation time and room-temperature quantum Hall effect, are mainly caused by the existence of so-called Dirac point and Dirac cone in the band structure^6^,^7^,^8^. However, a recent work shows that some allotropes of graphene also have Dirac cones, and they all belong to the family of graphyne^9^,^10^,^11^.

Graphyne is the allotrope of graphene, which contains both *sp* and *sp*^*2*^ hybridized carbon atoms^12^,^13^. There are many possible structures in graphyne family^12^,^14^,^15^,^16^, and recently, graphdiyne, a member of graphyne family, has been successfully synthesized^17^ and widely studied^18^,^19^. On the basis of graphdiyne, we believe that other families of graphyne might be realized experimentally soon after^20^,^21^,^22^. Among those graphyne structures, α-graphyne has caught our attention, which has honeycomb lattice structure and hexagonal symmetry like graphene^23^,^24^,^25^. Recently, the Dirac cones are also found in α-graphyne’s band structure in many researches^9^,^26^,^27^,^28^. Moreover, due to the presence of acetylenic bonds in the structure, the electonic and spintronic properties of α-graphyne nanoribbons (α-GYNRs) are thought to be have some differences from those of graphene nanoribbons (GNRs)^16^,^29^,^30^,^31^,^32^. Therefore, based on a great deal of valuable researches of GNRs, exploring the properties of α-GYNRs would be necessary.

As we know, due to the properties of spin polarization, zigzag graphene nanoribbons (ZGNRs) are more notable for their potential applications in spintronic devices such as spin-filter^33^,^34^, spin-valve^35^,^36^, giant magnetoresistance devices^37^,^38^, etc. Motivated by this, we mainly focus on the zigzag α-graphyne nanoribbons (Zα-GYNRs) now to see whether the spin polarization can be found, and the potential application value of them in spintronic devices. In this work, we study the electronics, spintronics and spin caloritronics properties of a particular Zα-GYNR, discussing the mechanism and designing several nanodevices based on the transport characteristics.

## Result

### The electronic structures of Zα-GYNRs

**Figure 1:**
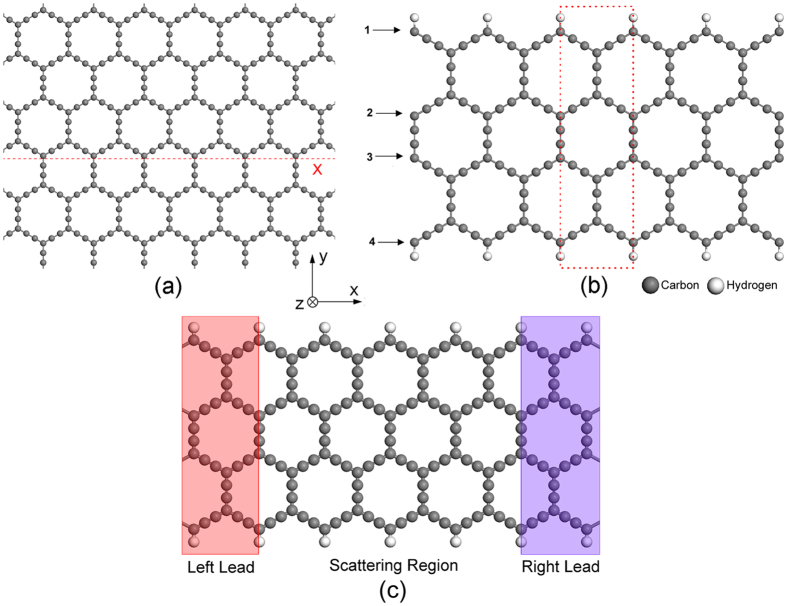
Structures of (**a**) two-dimensional infinite α-graphyne sheet and (**b**) one-dimensional α-graphyne nanoribbon cutting along x direction. (**c**) is A schematic device model of two probe system. The red frame indicates the semi-infinite left leads while the blue one indicates the semi-infinite right leads.

The structure of α-graphyne is shown in Fig. 1(a). This size of structure is stable and durable according to a stability research of α-graphyne recently^39^. In order to obtain Zα-GYNRs, we process the two-dimensional α-graphyne analogous to graphene^6^,^39^. We optimize the two-dimensional infinite α-graphyne sheet and cut it into one-dimensional nanoribbon along x direction. The terminations of the α-GYNRs are both zigzag carbon chains, and the width of the Zα-GYNR is determined by the number of zigzag carbon chains^40^. In our research, the width of the nanoribbon structure is 4-Zα-GYNRs (shown in Fig. 1(b)), and the dangling bonds at the edges of all the Zα-GYNRs are passivated by hydrogen atoms, in order to saturate the dangling bonds. Note that the possible structures of the Zα-GYNRs are much more than ZGNRs, and the edge configuration discussed in our paper is just a common and stable one.

**Figure 2:**
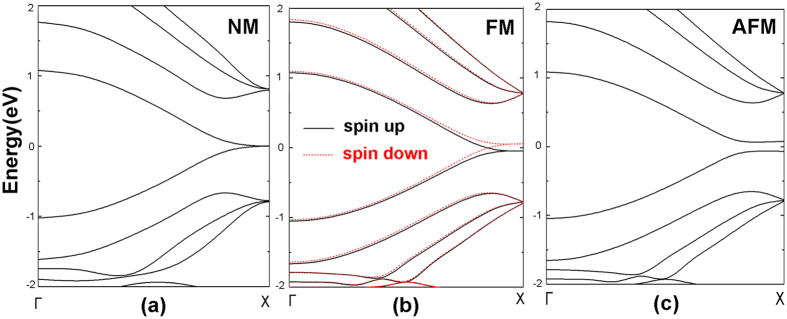
The band structures of (**a**) non-magnetic, (**b**) ferromagnetic and (**c**) antiferromagnetic Zα-GYNR.

As we know, the ZGNR has been reported having a magnetic insulating ground state with antiferromagnetic coupling of spin polarized edge states^41^, and can be magnetized by applying a sufficiently strong magnetic field, leading to a ferromagnetic alignment of spin polarized edge states^6^,^37^. In analogy with ZGNR, we calculated the band structures of Zα-GYNR in non-magnetic (NM), ferromagnetic (FM) and antiferromagnetic (AFM) states, which are shown in Fig. 2(a–c) respectively. It is obviously seen that in non-magnetic state the Zα-GYNR is metallic since the bands go across the Fermi level. In ferromagnetic state, the Zα-GYNR is also metallic but there appeares obvious spin splitting, the band structure of spin-up one moving up while the spin-down one moving down, leading to an effective magnetic moment. However, in antiferromagnetic state, the band structure opens a tiny band gap and the Zα-GYNR is a semiconductor without spin polarization. We have also calculated the energies of these three states, and the results show that in antiferromagnetic state the energy is the lowest while in ferromagnetic state the energy is the highest, so the antiferromagnetic state is the ground state of Zα-GYNR. These properties of Zα-GYNR are quite similar with ZGNR.

### The spin transport characteristics of Zα-GYNRs

**Figure 3:**
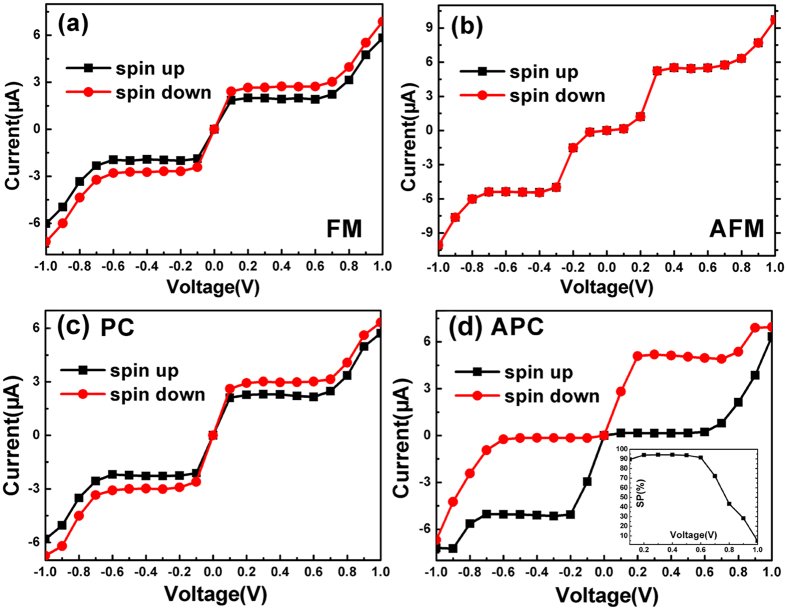
Spin-depended I-V curves for Zα-GYNR in (**a**) ferromagnetic, (**b**) antiferromagnetic state, (**c**) PC and (**d**) APC configuration. The inset of (**d**) shows the spin polarization in APC configuration as a function of bias voltage.

Then we study the transport properties of the Zα-GYNR. We construct the device as shown in Fig. 1(c), where the left and right electrodes are semi-infinite and the centre region is the scattering region. The transport direction is from left to right, and the temperatures of the electrodes are all set to 300 K. For FM and AFM states, the whole device is polarized, including the leads and the scattering region, and the calculated I-V curves of Zα-GYNR are shown in Fig. 3(a,b) respectively. In FM state, (Fig. 3(a)) the curves show obvious metallic transport properties and the current is generating as soon as the bias voltage is applied. When bias voltage goes from ±0.1 V to ±0.7 V, the interesting thing happens, that the values of currents nearly hold a constant and the curve looks like a platform. Whereafter, when the bias is larger than ±0.7 V, the currents grow quickly with the increasing bias voltage. Meanwhile, the currents show clear spin-splitting between the spin-up and spin-down ones, but the spin polarization is quite small since the effective magnetic moment is tiny. In the AFM state (Fig. 3(b)), there exists a threshold voltage at about ±0.1 V, which is caused by the band-gap of AFM state in band structure (Fig. 2(c)). When the bias reaches ±0.3 V, the current curve also looks like a platform until bias reaches ±0.7 V, very similar with the FM state. However, since there is no effective magnetic moment in AFM state, the spin-up and spin-down currents are totally coincide with each other, and no spin-splitting is shown. Note that the device in AFM state is used for comparison and have little practical application, since the leads in reality would best be a ferromagnetic metal.

In order to develop the spin-polarization, we adjust the magnetic direction and set the initial magnetization configuration of the device (shown in Fig. 1(c)) to parallel configuration (PC, the spin orientation of left and right electrodes are both up) and antiparallel configuration (APC, the spin orientation of left electrode is down and the right one is up). The scattering region is non-magnetic. The corresponding transport characteristic curves are shown in Fig. 3(c,d).

For PC configuration, we see that the I-V curves are quite similar to that of FM state (Fig. 3(a)), only the values of the currents are slightly smaller, which is caused by the absence of magnetic field in centre region. For APC configuration, the interesting transport behaviors attract our attention. We find that under positive bias, the spin-down current rises rapidly with the bias increasing, and forms an approximate platform from 0.2 V to 0.7 V. Meanwhile, the spin-up current keeps very little value in this bias region, and the curve almost can not be observed. When the bias passes 0.7 V, both the spin-up current begins to rise up rapidly, and intersects the spin-down curve at around 1.0 V bias. In addition, the curves of negative bias are diametrically opposed to the positive situation. Thus, spin-up and spin-down currents show obvious spin polarization in APC configuration, and the spin polarization curve is shown in the inset of Fig. 3(d). Note that the spin-polarization is calculated by SP = (I_up_ − I_down_)/(I_up_ + I_down_) × 100. We find that the spin polarization is quite high and steady at lower bias, which is up to 95%, but suddenly drop down at bias 0.7 V and finally approach zero at 1.0 V.

### The nanodevices based on Zα-GYNR

**Figure 4:**
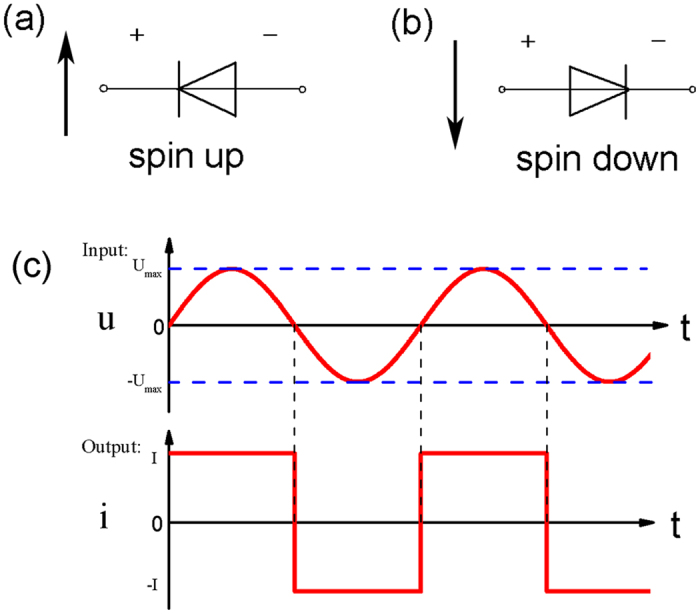
The diagram of the dual spin diode: (**a**) the reverse conducting diode for spin-up current, and (**b**) the forward conducting diode for spin-down current. (**c**) The diagram of the molecule converter of square signal.

Based on the transport properties shown above, we discuss the potential value of Zα-GYNR on spintronics device. In APC configuration (Fig. 3(d)), the Zα-GYNR can be used as dual spin-filter under finite bias voltages^34^. Since the spin-up and spin-down electrons are unidirectionally filtered, we can get spin-down current under positive bias and get spin-up current under negative bias. What is more, if the bias is controlled in a proper region, the output spin currents act approximately as a constant value. On the other hand, the Zα-GYNR also can be used as a dual spin diode in APC configuration. When the current is pure spin-up current, the device is equivalent to the reverse conducting diode like Fig. 4(a); when the current is pure spin-down current, the device is equivalent to the forward conducting diode like Fig. 4(b).

In FM state and PC configuration, we can design another nanodevice based on Zα-GYNR. Seen from the curves in Fig. 3(a,c), if the bias voltage (input) is controlled in a proper region, the value of the current (output) can be controlled nearly a constant. For example, if the bias voltage is controlled within ±0.7 V (without zero bias), the value of the total current will be kept around 5.2 μA, and the current direction is in accordance with the bias voltage. Based on the properties, this system of Zα-GYNR can be designed as a molecule signal converter of square wave. As we know, square wave is an important switching signal, whose high level and low level could be the “1” and “0” in digit signal, can successfully connect the analog circuit and digital circuit^42^. Figure 4(c) is a diagram of the input and output signal. If the input signal is finite and in a proper region, the output square wave signal can be generate, realizing a converting from sinusoidal wave or triangular wave to square wave. Note that this diagram is an idealization, since the current output can not be so perfect in instantaneous step and steady level, which needs rectification and filtering and other subsequent processes. On the whole, an embryonic form of molecule signal converter of square wave begins to take shape.

### Spin Seebeck effect and spin caloritronics device

**Figure 5:**
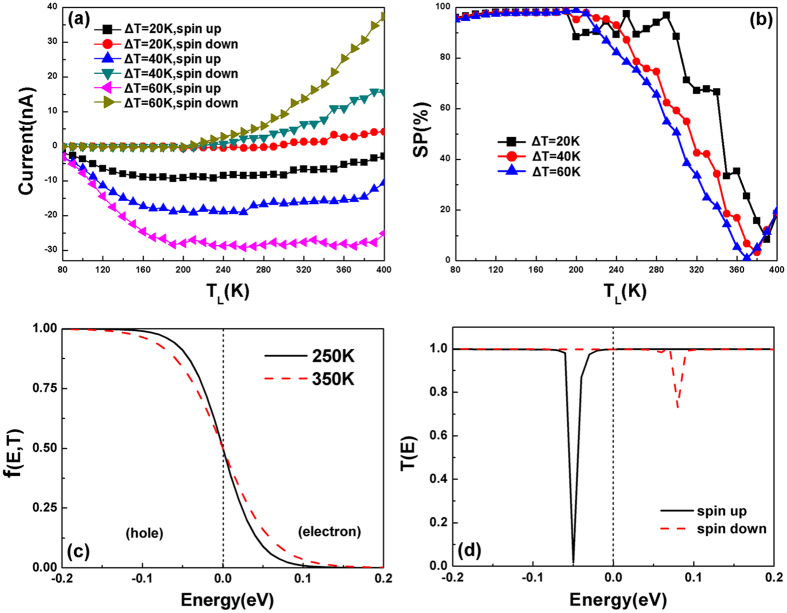
(**a**) The thermal induced spin-dependent currents; (**b**) the spin polarizations as a function of TL with three different ΔT; (**c**) The Fermi distribution of electrode under different temperatures; (**d**) the spin-resolved transmission spectrum of the device with zero bias voltage.

Besides the nanodevices mentioned above, we can also design spin caloritronics device^43^,^44^ based on the Zα-GYNR, whose appearance is the same as the device shown in Fig. 1(c), only the bias voltage is replaced by a temperature gradient. That means the bias voltage is zero and a temperature gradient is added to the device. We set the temperature of left electrode with T_L_ and the right one with T_R_ (T_L_>T_R_), so the thermally induced current is only caused by a temperature difference (ΔT = T_L_ − T_R_), and the magnetization configuration of the device is PC configuration. Figure 5(a) shows the calculated thermally induced currents versus T_L_ with different ΔT. It is clearly seen that the spin-polarized currents are generated without any bias voltage, wherein the spin-up current is negative and the spin-down one is positive. This is an obvious spin Seebeck effect since the spin-up and spin-down currents flow in opposite directions, which are generated only from a temperature gradient^45^,^46^,^47^. From the curves of the spin-depended thermal currents, we can see that that for T_L_ from 80 K on, there is no threshold temperature of spin-up current, but the threshold temperature of spin-down one is about 200 K, around which the spin-up ones reach the maximum value. Then, the spin-up current just decline slightly and almost keep stable, while the spin-down one increases rapidly from now on. Finally at 400 K, the values of spin-down currents are even larger than spin-up ones with each ΔT. It is notable that the values of SPs (SP = (|I_up_| − |I_down_|)/(|I_up_| + |I_down_|) × 100) of the thermally currents are quite remarkable (shown in Fig. 5(b)), which is quite high in a large region of temperature. Especially when the T_L_ is lower than 200 K, the SPs surpass 90% and keep almost stable. Simultaneously, we find that the changes of ΔT nearly have little effect on the SPs in low temperatures.

The thermally induced currents and spin Seebeck effect is caused by the interaction of the electrodes’ Fermi-Dirac distribution and the asymmetric spin-polarized transmission spectra. We plot the Fermi-Dirac distributions in a hypothesis situation that T_L_ = 350 K and T_R_ = 250 K for example, which is defined as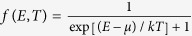, and the curves are shown in Fig. 5(c). Because of the temperature difference, the concentration difference of carriers is appeared. We know that the carriers are electrons (above the Fermi level) and holes (below the Fermi level), so both the electrons and holes would flow from left electrode to the right one, resulting in a negative electron current I_e_ and a positive hole current I_h_. If the transmission spectrum is symmetric, I_e_ and I_h_ will canceled each other, leading to zero net current. However, the actual spin-polarized transmission spectra of our device are asymmetric under zero bias, so the thermally induced spin-polarized currents are generated. Seen from Fig. 5(d), around Fermi-level, the transmission valley of spin-up one appears below Fermi level, so I_e_ > I_h_ and leading to a negative spin-up current. Conversely, the spin-down one shows transmission valley above the Fermi level, so I_h_ > I_e_ and we get a positive spin-down current. As a result, the Zα-GYNR device exhibits the spin Seebeck effect as shown in Fig. 5(a). What is more, it is known that the range of the Fermi distribution is determined by the temperature, and the thermal current will occur only when the Fermi distribution overlaps with the asymmetric transmission spectrum near the Fermi level^48^. We also notice that the spin-up transmission valley is closer to the Fermi level than the spin-down one, so when T_L_ = 80 K, the spin-up transmission valley is in the range of Fermi distribution while spin-down one is not. That is why spin-down current have a threshold temperature but spin-up ones have no in our result. When the T_L_ reaches 200 K, the Fermi distribution enters the transmission valley of spin-down one, so the spin-down current increases rapidly. But this time, since the region of Fermi distribution exceeds the spin-up transmission valley, the spin-up current achieves almost stable from now on (Fig. 5(a)). Note that the effect of the phonon is neglected in our calculation and we mainly focus on the electron transmission.

## Discussion

**Figure 6:**
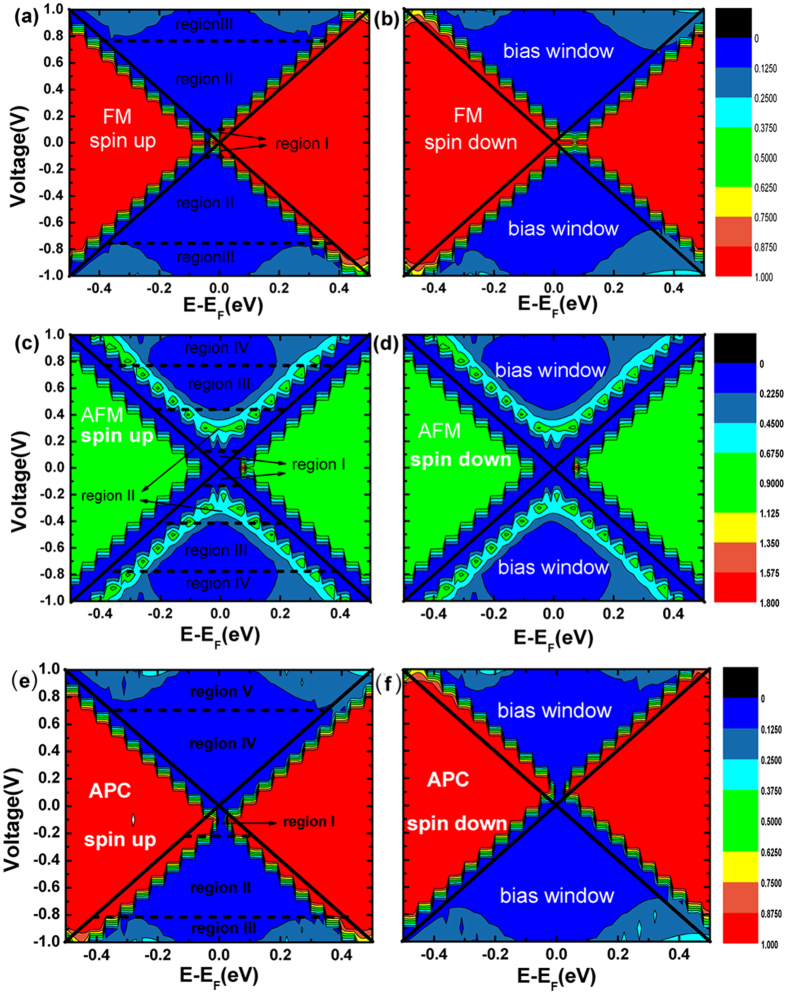
Spin-dependent transmission spectra as a function of electron energy E and bias voltage for (**a**) spin-up electron in ferromagnetic state, (**b**) spin-down electron in ferromagnetic state, (**c**) spin-up electron in antiferromagnetic state, (**d**) spin-down electron in antiferromagnetic state, (**e**) spin-up electron and (**f**) spin-down electron in APC configuration.

In order to explain the tansport behaviours especially the spin-polarizations and conductance plateaus (Fig. 3), we present the spin-resolved transmission spectra versus energy level and bias voltage in Fig. 6. The spin and magnetic configuration are labelled on the pictures. The intersecting solid straight lines are referred to the bias window, and From Eq. (1), we know that the current is determined by the corresponding integral area of the transmission coefficient within the bias window.

First, seen from the spin-up transmission spectra of FM state (Fig. 6(a)), when the bias is zero, the transmission coefficient of the transport channel is quite large at Fermi level, so the current increases rapidly once the bias is applied, and keeps growth when the bias window increases within region I (V < 0.1 V). However, the growth of the current is stopped immediately. When the bias keeps increasing, the peaks of the transmission spectra move away from the Fermi level and shift to the right side above Fermi level. Since the movement is proportional to the bias, the transmission channels are not increased in region II (from bias 0.1 V to 0.7 V.), and the currents nearly keep unchanged. When the bias is lager than 0.7 V in region III, although the peak of the transmission spectra keeps moving to the right side, there appear two new transmission channels in bias window, small transmission coefficient but quite broad channel width. That explains why the current increases rapidly when bias exceeds 0.7 V. The spin-down transmission spectra (Fig. 6(b)) have certain features in common with the spin-up one, but at variance in two respects: one is when the bias applied, the spectra movement directions are opposite, the spin-up one moves to right above Fermi level while the spin-down one moves to the left below Fermi level; the other one is the efficient transmission channels of spin-down electrons in bias window are slightly broader than spin-up ones, which leads to a slightly larger spin-down current in Fig. 3(a).

Next, let us see the AFM state of Fig. 6(c,d), the spin-up and spin-down spectra are exactly the same, so the currents show no spin splitting (Fig. 3(b)). When bias is zero, the transmission coefficient is so small and nearly no transport channel exists at Fermi level. When small bias is added, the transmission spectra retain small and the currents retain nearly zero within region I (Fig. 6(c)). Therefore we can see a semiconductor characteristic I-V curve, and the threshold voltage is about ±0.1 V (Fig. 3(b)). Then with the increasing of bias in region II, the transmission peak and effective transport channel appears in bias window, so the currents increase rapidly now. However in region III, the transmission spectra move to both sides away from the Fermi level and the transmission channel nearly unchanged, so the currents maintain a constant value from 0.3 V to 0.8 V. When the bias exceeds ±0.8 V, broader transmission peaks appear in the bias window in region IV, bringing in plenty of transport channels, and the currents increase immediately.

Then let us discuss the spin-resolved transmission spectra of APC. First see the spin-up spectra (Fig. 6(e)). Under negative bias, the bias window is divided into three regions. In region I, the transmission spectra and integral areas expand gradually with the bias increasing, so the current increases within −0.2 V. In region II, the spectra move to both sides of Fermi level, so the number of transport channels is not changed obviously, and the current almost remains constant although the bias increased. In region III, two new broad transport channels appear in the bias region, so that the current starts to increase again in this region. While under positive bias, the spectra are quite different and the bias window can be divided into two parts. In region IV, there is nearly not any spectrum and transmission channel in this region, so the current is so tiny and hard to observed in this region within about 0.6 V. In region V, plenty of transport channels appear and current increases rapidly in this region. It is apparently seen that the spin-down spectra are axisymmetric transversely with the spin-up spectra (Fig. 6(f)), so the behavior of I-V curve is diametrically opposed to the spin-up one.

The above discussion explained the behaviours of spin-polarized currents through the transmission spectra. Moreover, the origination of the transmission spectra should be related to the electron transmission between the two electrodes. Therefore, further studies are needed in our next discussion.

**Figure 7:**
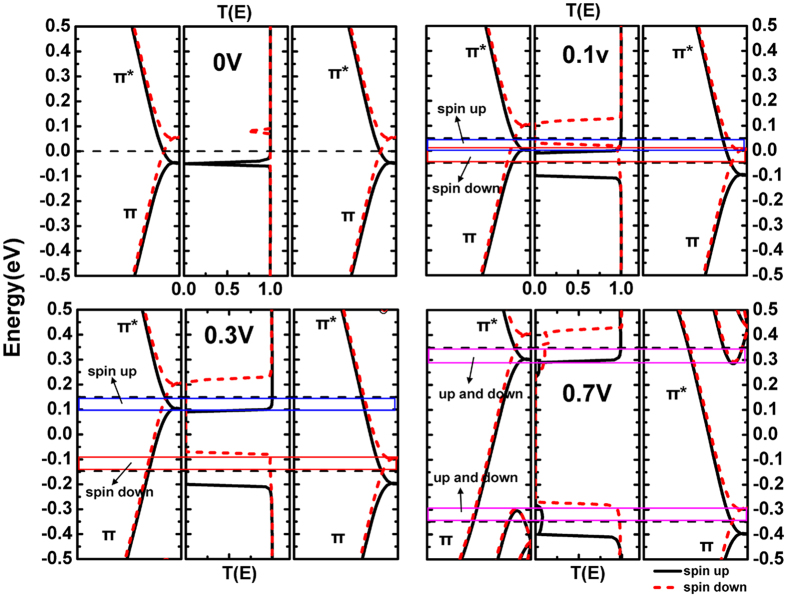
(**a–d**) In PC configuration, band structures of left electrode (left panel) and right electrode (right panel), along with the transmission spectra (middle panel), in bias of 0 V, 0.1 V, 0.3 V and 0.7 V separately. The dotted black line area represents the bias window and the colored rectangular frame represents the efficacious transmission channel.

**Figure 8:**
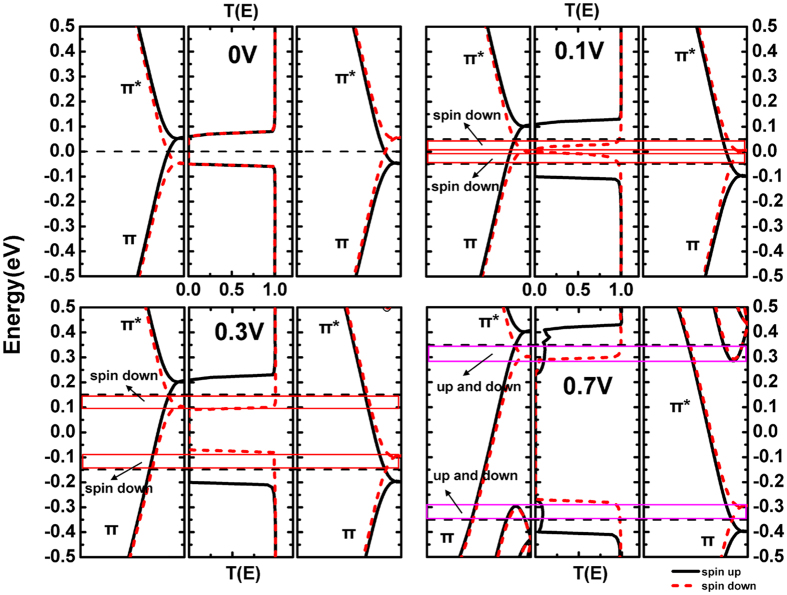
(**a–d**) In APC configuration, band structure of left electrode (left panel) and right electrode (right panel), along with the transmission spectra (middle panel), in bias of 0 V, 0.1 V, 0.3 V and 0.7 V separately. The dotted black line area represents the bias window and the colored rectangular frame represents the efficacious transmission channel.

We take the conditions of four typical bias (0 V, 0.1 V, 0.3 V and 0.7 V) and two magnetization configurations (PC and APC) for example. Each section of Figs 7 and 8 displays the band structures of left electrode (left panel) and right electrode (right panel) along with the transport coefficient (middle panel) in different conditions. One can find that only the bonding π and antibonding π* subbands appear near the Fermi level. We know that under theσmirror operation, the wave function of π subband has even parity while π* has odd parity of 4-Zα-GYNR^49^. Moreover, previous research found that when symmetry of spin subbands of the electrodes matches (the same), the transmission channel is open, and when mismatches (the opposite), the channel is closed^50^,^51^. Thereupon, from Figs 7 and 8 we find that the I-V characteristics and transmission spectra can be perfectly match with the symmetries of electrodes’ band structures.

In PC configuration, under 0 V bias (shown in Fig. 7(a)), since the spin orientation of left and right electrodes are both up, the spin-resolved band structures are exactly the same of both electrodes. Therefore, the symmetries of the band structures of left and right leads are certainly match to each other. The transmission channel is open and the transport coefficients are high enough except the deep but tiny dropdown at the contact of π and π* subbands, where the symmetry is disturbed. Especially, around Fermi level, the spin-up (spin-down) bands of left and right electrodes both have odd (even) parity, so spin-up (spin-down) transport channel is open and the coefficient is high at Fermi level.

When the bias is added to the electrodes, a positive (negative) bias makes the energy bands move up (down). The movement of the band structures, lead to a variation of the symmetry matching of left and right leads. For both spin-up and spin-down electrons in Fig. 7, when the left is π (π*) subbands but the right is π* (π) subbands, the symmetry is mismatch, the transport channel will be closed. Here three typical conditions (0.1 V, 0.3 V and 0.7 V) are shown in Fig. 7(b–d), and the bias window [V_L_, V_R_] is indicated by the dotted line area. The blue (red) rectangular frame represents the efficacious transmission channel of spin-up (spin-down) electrons within the bias window, which has a practical effect to the spin-depended transmission, and the purple rectangular frame represents the channel is open for both spin-up and spin-down electrons.

Seen from Fig. 7(b), because of the movements of band structures, the symmetry is changed within the bias window. Take the spin-up condition for example, within the blue rectangular region, left and right subbands have the same symmetry (odd), so the transport channel is open; in the rest region of bias window, left subband (even) and right subband (odd) have opposite symmetry, so the transport channel is closed. For the spin-down condition, the efficacious transport channel is in red rectangular region. When the bias voltage increases to 0.3 V (Fig. 7(c)), the bias window enlarges, and the movements of subbands keep going on. Because of the symmetry matching, the range of efficacious transmission channels (blue and red rectangular frame) are almost unchanged. This situation lasts from 0.1 V to 0.6 V, and as a result, the spin-dependent current keeps almost a constant in this bias region (Fig. 3(c)). When the bias reaches 0.7 V, the situation changes, which is shown in Fig. 7(d). Some other subbands enter the enlarged bias window and bring a large number of transmission channels, so the transmissions are not just depends on the symmetry of π and π* subbands, and the current increases rapidly from now on (Fig. 3(c)).

In APC configuration and under 0 V bias, since the spin orientation of left electrode is down and the right one is up, the spin-resolved band structures are changed and shown in left and right panel of Fig. 8(a). Around Fermi level, for spin-up situation, left subband (π, even) and right subband (π*, odd) have opposite symmetries, so the spin-up transport channel is closed; for spin-down situation, left subband (π*, odd) and right subband (π, even) also have opposite symmetries, so the spin-down transport channel is closed too. Therefore, a zero transmission gap (ZTG) can be seen in the transport spectrum (Fig. 8(a) middile panel).

When the bias voltage is added, the movements of the left and right band structures lead to two diametrically different consequences to spin-up and spin-down transport channels. When the bias is 0.1 V (Fig. 8(b)), the spin-up ZTG enlarges but spin-down one shrinks since the different symmetries of left and right subbands. Therefore, in bias window, the spin-up channel is closed and spin-down one is open. The effective transmission channels are all for spin-down electrons, and the spin-up current can not be observed (Fig. 3(d)). When the bias is 0.3 V (Fig. 8(c)), all the spin-up and spin-down ZTGs are enlarged. Still not any spin-up channel can be seen in the bias window while the efficacious spin-down transmission channels are nearly unchanged. Actually, this situation lasts from bias 0.2 V to 0.6 V, so only spin-down current can be obtained and almost remains stable in this region (Fig. 3(d)). When the bias reaches 0.7 V (Fig. 8(d)), for both spin-up and spin-down electrons, some other subbands enter the enlarged bias window, so a large number of transmission channels are brought in. Consequently, both spin-up and spin-down currents increase rapidly from now on.

Thus, by discussing the the symmetry matching of left and right electrodes’ band structures, we successfully explained the different transport behaviors and transmission spectra in PC and APC magnetization configurations. The discussions of FM and AFM magnetic states are similar and not presented here. Note that our research is concerned with pure zigzag α-graphyne nanoribbons and not attached to the metalic surface, since the ferromagnet may induce changes in the bandstructure of graphyne^52^.

## Method

Our first-principles calculations are based on the ATOMISTIX TOOLKIT (ATK) package, which adopts spin density functional theory combined with nonequilibruim Green’s function^53^,^54^,^55^. The core electrons are described by norm-conserving pseudopotentials, and the local-density approximation (LDA) is used for the exchange-correlation potential^6^,^56^. A single-polarized basis set is used and the cutoff energy is 150 Ry and a Monkhorst-Pack k-mesh of 1 × 1 × 100 is chosen in our work. The convergence parameters of the optimization were chosen as follows: total energy tolerance 1 × 10^−5^ eV/atom, maximum force tolerance 0.05 eV/Å. The vacuum layers between two sheets are larger than 15 Å. The NEGF-DFT self-consistency is controlled by a numerical tolerance of 10^−4^ eV. The spin-dependent current through the system is calculated using the Landauer formula:
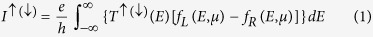
where *f*_*L*(*R*)_(*E*, *μ*) is the equilibrium Fermi distribution for the left (right) electrode, and *μ*_*L,R*_ = E_F_ ± *eV*/2 is the electrochemical potentials of the left and right electrodes in terms of the common Fermi energy E_F_, and T^↑(↓)^(E) is the spin-resolved transmission defined as
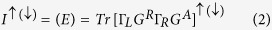
where G^R(A)^ is the retarded (advanced) Green’s functions of the central region and Γ_L(R)_ is the coupling matrix of the left(right) electrode.

## Additional Information

**How to cite this article**: Ni, Y. *et al*. The spin-dependent transport properties of zigzag α-graphyne nanoribbons and new device design. *Sci. Rep.* **6**, 25914; doi: 10.1038/srep25914 (2016).
